# The impact of indoor air pollution on children’s health and well-being: the experts’ consensus

**DOI:** 10.1186/s13052-024-01631-y

**Published:** 2024-04-14

**Authors:** Elena Bozzola, Rino Agostiniani, Laura Pacifici Noja, Jibin Park, Paolo Lauriola, Tiziana Nicoletti, Domenica Taruscio, Giovanni Taruscio, Alberto Mantovani

**Affiliations:** 1 Pediatric Unit, Bambino Gesù Children’s Hospital, IRCCS, Rome, Italy; 2 The Italian Pediatric Society, Rome, Italy; 3 Saint Camillus International University of Health Sciences, Rome, Italy; 4 Rete Italiana Medici Sentinella per l’Ambiente (RIMSA), ISDE/FNOMCeO, Rome, Italy; 5 Association of the chronically ill and rare patients, Cittadinazattiva APS, Rome, Italy; 6 Study Center KOS-Science, Art, Society, Rome, Italy

**Keywords:** Children, Indoor environment, Environment and health, Indoor air pollution, Mortality, Morbidity, Prevention, Risk analysis, Exposure, Risk reduction

## Abstract

**Background:**

Pollution of the indoor environment represents a concern for human health, mainly in case of prolonged exposure such as in the case of women, children, the elderly, and the chronically ill, who spend most of their time in closed environments.

**Main body:**

The aim of the study is to organize a group of experts in order to evaluate the evidence and discuss the main risk factors concerning indoor air and the impact on human health as well as challenging factors regarding preventive strategies to reduce pollution. The experts highlighted the main risk factors concerning indoor air, including poor ventilation, climatic conditions, chemical substances, and socio-economic status. They discussed the impact on human health in terms of mortality and morbidity, as well as challenging factors regarding preventive strategies to reduce pollution.

**Conclusion:**

The experts identified strategies that can be reinforced to reduce indoor pollution and prevent negative consequences on human health at national and local levels.

## Background

Pollution is becoming day after day an alarming threat to our planet. The international and national public health agencies have significantly focused on addressing food pollution, particularly concerning pesticide residues and bioaccumulating substances. Additionally, they have directed considerable efforts towards combating outdoor atmospheric pollution, stemming from emissions in urban areas, industrial facilities, and vehicle exhaust. Meanwhile, whereas people in high-income Countries (HICs) spend much of their lives indoors, the pollution of the indoor environment has still to receive due attention [1–3]. Indeed, domestic air and indoor pollution can be traced back to prehistory, when humans first moved to temperate climates, started building shelters, and used fire for cooking, heating, and lighting.

Indoor pollution is a global health issue. Today around the world, around 2.4 billion people still cook using solid fuels (such as wood, agricultural waste, coal, and manure) and kerosene on fires or stoves. Most of these people are poor and live in low- and middle-income countries with a large discrepancy between urban and rural areas. In 2020, only 14% of individuals residing in urban areas depended on polluting fuels and technologies, starkly contrasting the 52% prevalence observed among the global rural population [4]. 

Despite transitioning from biomass fuels to petroleum products and electricity accompanying modernization in developed countries, pollution remains a persistent threat to public health [5]. 

Indoor pollution is generated by the use of inefficient and polluting fuels, technologies, and/or materials at home and in closed spaces where people spend most of their time, including schools, healthcare facilities, gyms, and entertainment structures (cinemas, museums, etc.). Indoor air can be even more polluted than outdoor air when ventilation is inadequate and/or when heat and humidity facilitate the concentration of allergens, infectious agents, dust, etc [3, 6]. Besides, exposure to indoor pollutants can be greater than outdoor also because the amount of time people spend inside confined environments may be greater [3]. Therefore, whereas exposure is a problem for everyone, the concern is even greater for women, children (e.g. schools), the elderly (e.g. retirement homes), and the chronically ill, who spend most of their time at home and in other closed environments; moreover, indoor pollution is greater in relation to lower socio-economic status [4, 7]. 

The main exposure to indoor pollutants is through inhalation; nevertheless, it is crucial to consider cutaneous and oral exposure, particularly among children who engage in activities that involve contact with floors and frequently exhibit hand-to-mouth interactions [8, 9]. Wilson’s study revealed that children touch their mouths, eyes, and nose more frequently than adults. In particular, hand-to-mouth contact may be a matter of concern when considering exposures to chemicals, such as lead or pesticides [8]. 

## Main text

The KOS Study Center - Science, Art, Society organized the working table “Living and working in healthy environments: indoor air, construction materials, furniture” to draw attention, evaluate the evidence, and discuss future perspectives regarding the impacts on human health and well-being of the quality of indoor environments in which the majority of people spend time.

An interdisciplinary group of experts highlighted the complexity of the indoor environment. The experts have been selected based on their level of expertise and professional experience in indoor pollution or as delegates from Italian Scientific Societies or Associations. An electronic search was undertaken on the PubMed database, using all of the important concepts from our basic clinical question, avoiding unnecessary filters. So, the terms “air pollution indoor” and “air quality, indoor” have been used as Mesh Terms, limited to humans and English reports. The database was shared and the objectives of the consensus were discussed on September 30th, 2023. Since then, each expert has independently analyzed the texts and their references. On December 11th, 2023 the experts discussed and highlighted the main risk factors concerning indoor air and the impact on human health as well as challenging factors regarding preventive strategies to reduce pollution.

Accordingly, the three identified topics examined are: identification of the main risk factors for indoor air and environment; investigation of the impact of indoor pollution on human health; and highlighting challenging factors regarding prevention and health risk reduction.

### Main risk factors concerning indoor air and environment

Certain individual risk factors are rather well-recognized, including infections via air conditioning, allergens, radon, and passive tobacco smoking, yet, the scenario of indoor risk factors is far more complex and calls for a comprehensive appraisal. The group of experts identified the main risk factors for the quality of indoor air which require further evaluation: poor ventilation, climatic factors, chemical substances, and low socio-economic status.

#### Poor ventilation

In poorly ventilated areas, fine particle levels may be 100 times higher than acceptable levels and may result in respiratory diseases such as asthma, allergy, and sick-building symptoms. In addition, improving on indoor ventilation system reduces the humidity, thus inhibiting the growth of microbes [4, 10, 11]. Monitoring the maintenance of the ventilation system is essential to ensure proper functionality, as dirty filters and blocked vents can impede the circulation of air [12]. The problem is of concern especially if we consider school buildings, where children spend up to 8 h a day in classrooms, at least between the ages of 6 and 19 years, and in many cases between 3 and 4 and 19 years. The conditions are often unacceptable and regardless of the geographic situation, all the current studies report similar problems: classrooms too small for the number of children in school classes, resulting in densely crowded rooms, as well as poorly conceived and managed ventilation [13]. Classrooms generally accommodate a large number of people and therefore require a certain air exchange in order to maintain low levels of carbon dioxide and other pollutants, as well as to allow children to spend a comfortable and profitable school time. Nevertheless, high carbon dioxide concentrations in classrooms, which indicate poor ventilation conditions, have been identified as the primary causes of poor indoor air quality in schools [13]. A good system of ventilation is a key factor. Evidence suggests that the level of exposure to indoor pollution is lower in classrooms placed on higher floors of buildings, which is likely due to better ventilation [14]. 

#### Climatic conditions

The ongoing and progressive changes in climate and ambient air pollutant concentrations have a significant impact on the quality of indoor air and the way people live in all regions of the world, especially on children due to their vulnerability. Climatic factors, including temperature and humidity, can affect the quality of indoor air as well as increase the risk of viral, bacterial, and fungal contamination [10, 15]. The study by Fan investigated that indoor fungal contamination is highest in the summer. This derives researchers into a concern about the population living in the tropical region. In turn, this event may elicit an increased use of fungicides, insecticides, and biocides [16]. Moreover, environmental alteration, such as land reduction, deforestation, etc., facilitates poor climatic conditions in the indoor environment as well as outdoor-to-indoor exposure to harmful emissions and infectious agents [17]. 

#### Chemical substances

Indoor environments present a large quantity of materials, with the presence of additives in paints, glues, fabrics, coverings, furnishings, electronic equipment, and more. Additionally, the prevalence of recycled materials in concrete contributes to the complexity of indoor settings. For example, indoor exposure to benzene (from glues, paints, and solvents) can be even higher than that in industrial environments; the same applies to carcinogenic formaldehyde used as a preservative in woods and textiles [18]. Among these substances, endocrine disruptors (ED) are particularly dangerous for children and pregnant women, as they are linked to reproductive and developmental disorders. In children, epidemiological studies suggest that ED may adversely affect prenatal and post-natal growth, thyroid function, glucose metabolism, body composition, and risk of obesity, puberty onset, and successive fertility through several mechanisms [19, 20]. These substances (including parabens, phthalates, bisphenols, and perfluoroalkyl substances) may be used as preservatives, biocides, and water repellents. For example, polybrominated chemicals have been widely used as flame retardants, while triazoles (steroid synthesis inhibitors) are fungicides intended for wood preservation. Substances identified as ED are subject to restrictions, but they can persist in materials and consequently in indoor dust [21]. For example, the exposure to polybrominated diphenyl ethers used in paints, plastics, foam furniture padding, textiles, rugs, curtains, televisions, building materials, airplanes, and automobiles, banned since 2006, may cause concerns for human health. In fact, due to their long persistence, they may leak from products over time and accumulate in the environment and humans for many years: several toxicological and epidemiological studies (also recently reviewed by the European Food Safety Authority (EFSA) indicate that the PBDE body burden is associated with adverse reproductive and neurobehavioral effects, especially in the developing organism; main modes of action are the disruption of thyroid and steroid hormone metabolism as well as oxidative stress [22–24]. 

#### Low socio-economic status

Data published by the World Health Organization (WHO) show that poverty can exacerbate the harmful health effects of air pollution by limiting access to information, treatment, and other health resources. For example, considering fuels and polluting technologies for cooking, the problem is above all in low- and middle-income countries. It is a matter of concern for 83% of the population in the African Region, 59% in the South-East Asia Region, and 42% in the Western Pacific Region. In the Americas and the European Region numbers are significantly lower, 13% and 6% respectively. Nevertheless, even in the WHO European Region, more than 1 out of 20 people is exposed to polluting cooking technologies [1]. 

Besides poverty, lower economic status with outdated, inadequate housing devices may represent one of the main sources of pollution in HICs. For example, in the European Union, cookers can produce much higher levels of indoor nitrogen dioxide pollution than outdoor ones. They can also cause carbon monoxide pollution. Replacing a gas stove with an electric one can decrease the median ambient nitrogen dioxide concentration by 51% [25, 26]. Cooking methods may influence indoor air quality (for example, roasting meat produces more PM2.5 than boiled food); in addition, also the dimension of the kitchen is an essential factor [27, 28]. The concentration of pollutants resulting from cooking the same meal is greater in a small environment rather than in a large kitchen [28]. 

### The impact of indoor pollution on human health

The experts focused on the effect of indoor pollution on both mortality and morbidity:

#### Mortality

Environmental pollution in 2019 had been responsible for approximately 9 million deaths per year, corresponding to one in six deaths worldwide [29]. In detail, according to WHO, the combined effects of ambient air pollution and household air pollution are associated with 6.7 million premature deaths annually [30]. Of note, 2.4 billion people worldwide (approximately one-third of the whole population) cook by open fires or inefficient stoves fuelled by kerosene, biomass, and coal, which are responsible for household air pollution [4]. Household air pollution was responsible for an estimated 3.2 million deaths per year in 2020, including over 237,000 deaths of children under the age of 5.

#### Morbidity

Adverse effects of indoor pollution may be traced to respiratory and non-respiratory systems. In the literature, there is strong evidence that children should be considered as a susceptible group as they are more susceptible to the health effects of air pollution than adults. The functionality of children’s immune and respiratory systems is still developing, hence increasing the vulnerability to exposure to environmental pollutants in indoor air and dust. Exposure can occur in diverse scenarios and settings. Apart from home, they can be exposed to indoor air pollution also in nursery and primary schools; in these settings, there may be a continuum between indoor and outdoor pollution, e.g., through the journeys to school and school playgrounds [3]. Indeed, nearby traffic is a key determinant of pollutant concentrations outside schools, which is relevant to indoor exposure through outdoor-indoor exchanges [3]. Moreover, at school, children are frequently more physically active than at home, increasing the ventilation rate and consequently the inhaled dose of pollutant concentrations [31]. Exposure to traffic-related air pollutants at school can also adversely impact neuropsychological development, in particular cognitive development, mainly memory and attention, as observed in primary school children [32]. Exposure to household air pollution nearly doubles the risk of childhood lower respiratory tract infections and is responsible for increased risk of non-communicable diseases, including stroke, ischemic heart disease, chronic obstructive pulmonary disease, and lung cancer [33–36]. A meta-analysis of 41 studies has shown that children living in a home with a gas stove have a 32% increased risk of developing asthma attacks [37]. Finally, indoor air pollution can contribute to aggravating airborne infections. Of note, an association between COVID-19 infection and pollution has been described during the pandemic period [38]. As mentioned before in the case of ED, adverse effects of indoor pollution are not limited to the respiratory system. For example, there is a connection between pollution and weight gain [35]. The main pollutants involved in this process are nitrogen oxides, nitrogen dioxide, ozone, and particulate matter, including PM10 and PM2.5 [26, 35, 39]. 

Indoor pollution may constitute a risk factor for future generations, inducing negative effects on health even before birth. Both prenatal and postnatal exposure to air pollution can negatively affect neurological development, lead to poorer cognitive test results, and influence the development of behavioral disorders such as autism spectrum disorders and attention deficit hyperactivity disorder. In particular, the role of pollution in an increased risk of neurodevelopmental disorders, anxiety, and depressive disorders even before birth has been highlighted [40–42]. As well, there is also a link between household air pollution, low birth weight, and reduced respiratory lung function [41]. 

### Challenging factors regarding prevention and health risk reduction

Indoor contaminants are invisible and odorless, so they can be defined as unknown enemies. In most cases, people are unaware of some chemical, biological, and physical products that are present inside houses. Scarce attention by public health agencies and policy makers as well as inadequate information and knowledge lead to underestimating the problem with long-term effects on the entire community. Of note, attention is present to isolated specific factors, but a comprehensive framework for primary prevention and risk analysis is lacking [15, 43]. 

In 2014, WHO issued the first guidelines on clean fuels and technologies for cooking, heating, and home lighting. The document aimed to guide policymakers and specialists working on energy and resources to implement the best strategies against household air pollution. Experts highlighted the importance of expanding the use of clean fuels and technologies including solar energy, electricity, biogas, liquefied petroleum gases, natural gas, alcohol fuels, as well as biomass stoves in line with the emission targets by WHO guidelines. Overall, while the WHO Guidelines need to be updated in order to build a comprehensive concept of the complex indoor scenario, their indications still retain their full validity. It is, therefore, necessary to rethink the settlement models in order to place living and the well-being of the person at the center of urban planning and housing [26].

Studies on indoor pollution should be increased to expand the knowledge and reduce remaining uncertainties. In particular, it is necessary to delve deeper into how the various pollutants circulate, how they interact with each other, and how they are influenced by climate change and other environmental drivers. These actions are consistent with the United Nations Agenda 2030 for sustainable development and for addressing inequalities in terms of health. A global health strategy and a transdisciplinary One Health approach are recommended for the integrated protection of all living beings and of the environment: conceivable and feasible regulatory measures for primary prevention include enforcing evidence-based limits for unavoidable pollutants and replacing hazardous substances and technologies. At a national and local level, in any home and indoor environment, through awareness raising and empowerment, it is possible to implement corrective strategies such as minimizing the lighting of fireplaces, using extractor hoods while cooking to avoid fumes and steam, clean air conditioning filters regularly [44]. 

## Conclusion

At a national and local level, in any home and indoor environment, strategies should be reinforced to reduce indoor pollution and prevent negative consequences on human health, requiring risk analysis, institutional commitment, and funding as well as the necessary involvement of policymakers.

The main actions that the expert group has identified are:

In conclusion, the experts agree on the urgent need for an approach to indoor air and the environment from a health risk assessment and management perspective, advocating policymakers and health care providers in finalizing strategies to reduce indoor pollution. Medical training as well as epidemiological and clinical studies should strengthen and disseminate the knowledge on indoor pollution and its health effects. Concrete directions and anticipatory guides may include scientific reports as well as awareness-raising campaigns on indoor air and environment; campaigns should be addressed to the general population, and children-tailored initiatives should be considered as well. These actions work on a cascade with a final desirable positive effect on the health of the population.

In detail, the suggested actions of policymakers, medical doctors, and the population are:

1. Publishing periodic regional and national updates of indoor pollution levels using appropriate indicators. There are variable national and regional limit values for gaseous substances and airborne particulate matter in the built environment, including schools, homes, healthcare facilities, and other public spaces. Moreover, indoor spaces are characterized by complex air chemistry, and their construction materials and types of activities vary significantly. Hence, a harmonization effort should be done to avoid unjustified differences in limit values based on up-to-date evidence and reasonable precaution.
2. Integrating local health networks. The indoor environment is variable, dynamic, and complex, considering the problems of solid–gas partitioning onto surfaces and particles, as well as the release and distribution of particles from different materials. Science-based models and approaches will help in order not to “drown in complexity”. Meanwhile, models and approaches for research on indoor air and environment are not harmonized, therefore, the opportunity for cross-study comparisons is missed. Indoor air chemistry is subject to specific boundary conditions; the required guidance documents for indoor-specific pollutants need to be developed in collaboration with local health networks. In general, interdisciplinary studies carried out through harmonized, comparable approaches by local health networks will be important to support and/or update specific regulations.
3. Creating a permanent national task force for the epidemiological surveillance of the effects of indoor pollution and the definition of risk analysis and health promotion strategies by timely actions.
4. Organizing training experiences based on local needs, with the contribution of civic organizations.
5. Planning preventive and corrective interventions, including redevelopment of unhealthy buildings and polluted areas, implementation of energy efficiency of the real estate assets (public and private), selection of low-hazard materials, renaturalization, and incentives for the greening of surfaces at homes, schools, and workplaces.
6. Providing sentinel doctors who can provide regular and standardized information by using appropriate indicators, and timely organized interventions as well as punctual and effective preventive actions.
7. Training on environment and pollution during medical degree courses and other health (e.g. prevention technicians) and non-health (e.g. engineering, architecture) faculties.
8. Medical training to promote awareness and knowledge of the genesis, extent, and modalities of the effects of the indoor environment on the health of the current and future generations, through the knowledge of environmental, social, and cultural determinants and their interactions.
9. Encouraging epidemiological and clinical research on the age- and gender-specific effects of indoor pollution, advocating national scientific studies. A large body of scientific studies has shown that exposure to indoor air pollutants has adverse effects on children’s health, both in the short term and long term, including reduced cognitive function, heart and respiratory disturbances, as well as increased predisposition to stroke, chronic obstructive pulmonary disease, and lung cancer. For this reason, increasing knowledge on indoor air pollution is helpful to promote public health, reduce de community disease burden as well as support policymakers in developing management approaches to the indoor environment from a health risk management perspective.
10. Information, awareness, and education of the population through family doctors and pediatricians. They can play a crucial role in increasing awareness, knowledge, and empowerment, promoting actions to preserve human health. Other professional categories, such as pharmacists, surveyors, construction workers unions, and condominium administrators, as well as civic organizations, should play a key role in spreading information. The involvement of the school system should be kept in mind in order to aware the new generations.

### Limitations

It should be mentioned that the chosen database (PubMed) focused on peer-reviewed literature, not including grey literature, such as working papers, opinions, and reports. The work team was chosen to identify available national experts, not including international ones, and did not face policymakers.

## Data Availability

Not applicable.

## Abbreviations

ED: endocrine disruptors
WHO: World Health Organization
